# Exploring the power of plants to overcome environmental stresses

**DOI:** 10.1186/s12284-014-0037-y

**Published:** 2015-02-12

**Authors:** Motoyuki Ashikari, Jian Feng Ma

**Affiliations:** Nagoya University, Furo-cho, Chikusa-ku, Nagoya 464-8601 Japan; Research Institute of Plant Science and Resources, Okayama University, Chuo 2-20-1, Kurashiki, 710-0046 Japan

Global environmental changes (GEC) have become a threat for crop production. GEC causes drought, flooding, soil acidification, soil salinization, extremely low and high temperatures, and other adverse environmental conditions. All of these stressful factors affect both crop yield and quality, directly or indirectly. It is also estimated that the world population will reach nine billion by 2050, therefore there is a demand to increase crop production to feed an additional two billion people during the next 40 years. Approximately 50% of the total calories consumed by humans are provided by the three major crops of rice (23%), wheat (17%) and maize (10%). It is especially necessary, therefore, to increase the production of these three crops to solve food shortages. Given that increase of land suitable for agriculture cannot be expected in the future, producing more of the desired products per unit area of land will be an important task. There are a number of approaches for increasing crop yields per unit, but one of them is definitely to enhance crop tolerance to various stresses caused by GEC.

Plants are very tough and have developed strategies to adapt to various environments during their long evolutionary process. Therefore, understanding the molecular mechanisms underlying stress tolerance is a prerequisite for enhancement of crop stress tolerance. In 2010, the Ministry of Education, Culture, Sports, Science and Technology (MEXT) of Japan launched a project entitled “**Integrated Analysis of Strategies for Plant Survival and Growth in Response to Global Environmental Changes**” to address this issue. The objective of this project is to systemically elucidate the power of plants to overcome environmental stress at the molecular level and to comprehensively understand the molecular networks involved, by bringing together researchers from different fields. This project consists of three work packages, namely, “Survival Strategy”, “Growth Strategy” and “Mathematical Modeling”. The outputs from this project will contribute to protecting the global environment and ensuring sustainable food production through breeding plants (crops) with enhanced stress tolerance.

Rice is an important staple food, which feeds nearly half of the world’s population. Although there has been great increase in rice yield during the past decades, there is still high potential to increase the yield by the introduction of new varieties with high yield and high stress tolerance. In this special issue, several important issues relating to stress tolerance in rice have been addressed. Cho and Oki describe the application of temperature, water stress, and CO_2_ in rice growth models. Todaka et al. review the dehydration-responsive element binding protein 1 (DREB1), which is an important transcription factor for drought and other stress tolerance. Ye et al. gave a review on the latest ABA signaling study under stress condition. Nishiuchi et al. and Horie et al. summarise recent understandings of water and salt stress in rice, respectively. Uraguchi et al. update our knowledge on Cd accumulation in rice, which is an important issue for food safety. We hope that these articles will help in better understanding of the mechanisms for stress tolerance in rice.

